# Serum from patients with hepatitis E virus-related acute liver failure induces human liver cell apoptosis

**DOI:** 10.3892/etm.2013.1398

**Published:** 2013-11-11

**Authors:** FAN WU, MINXIN WANG, DEYING TIAN

**Affiliations:** Department of Infectious Disease, Tongji Hospital, Tongji Medical College, Huazhong University of Science and Technology, Wuhan, Hubei 430030, P.R. China

**Keywords:** acute liver failure, serum, apoptosis, core polysaccharide

## Abstract

The pathogenesis of acute liver failure has not been fully elucidated. The present study investigated the effects of the serum from patients with hepatitis E virus (HEV)-related acute liver failure on human liver cell survival and apoptosis, and evaluated the protective effects of anti-lipopolysaccharide(LPS) antibody recognizing core polysaccharide against acute liver failure serum-induced apoptosis. Serum was collected from patients with HEV-related acute liver failure. The levels of endotoxin (LPS) in the serum were measured using a quantitative tachypleus amebocyte lysate endotoxin detection kit with a chromogenic endpoint. Serum with a mean concentration of LPS was incubated with L02 human liver cells and the rate of apoptosis was detected by flow cytometry. The apoptotic rate was also evaluated in liver cells incubated with antibody and the HEV-related acute liver failure serum. The results indicated that the concentration of LPS in the serum of patients with HEV-related acute liver failure was 0.26±0.02 EU/ml, which was significantly higher than that of the control group (P<0.05). The rate of apoptosis in the human liver cells induced by acute liver failure serum was 5.83±0.42%, which was significantly increased compared with that in the cells treated with the serum of healthy individuals (P<0.05). The apoptotic rate of the cells incubated with antibody and the acute liver failure serum was 5.53±0.51%, which was lower than that of the cells incubated with acute liver failure serum alone (P>0.05). These results indicate that the serum of patients with HEV-related acute liver failure induces the apoptosis of human liver cells. LPS may be directly involved in the apoptosis of human liver cells. Moreover, the presence of the antibody did not significantly reduce the level of apoptosis of liver cells exposed to HEV-related acute liver failure serum.

## Introduction

Acute liver failure is a significant clinical syndrome in which a previously normal liver fails within days or weeks. The prognosis of patients with acute liver failure remains poor and the overall mortality rate is 90% (1). At present, there are no effective treatment therapies for this disease and its complications result in a high mortality rate and resource cost (2–4). In the developing world, viral infections are predominant, with hepatitis E infection recognized as a common cause of mortality in many countries (5–8). The pathogenesis of acute liver failure has not been fully elucidated and the apoptosis of liver cells is an important event in the development of acute liver failure (9,10). It has been demonstrated that the serum from patients with liver failure may induce cytotoxicity and oxidative stress of HHY41 cells, and reduce DNA synthesis, protein synthesis and cytochrome P4501A activity (11). However, the effects of acute liver failure serum on liver cell survival and apoptosis and the underlying mechanisms have not been fully elucidated.

Endotoxin [lipopolysaccharide (LPS)] syndrome is a particularly grave complication since bacterial infection is confirmed in up to 80% of patients with fulminant hepatic failure (12–14). The association of liver injury with endotoxemia has been confirmed in a variety of experimental animals (15,16). Endotoxin syndrome is a systemic inflammatory response mediated by several of the cytokines produced by lymphocytes and macrophages (17–19), which exacerbates the disease condition of acute liver failure (20). LPS is significant in the development of liver failure (21).

The treatment of endotoxemia in liver failure is an important research area. Antibodies against LPS are considered to provide direct protective effects on endotoxemia, however, the anti-endotoxin effects of antibodies against lipid A have not been found to be satisfactory and the mechanisms of the protective effects have not been elucidated (22). Therefore, the present study focused on the effects of anti-LPS antibody recognizing core polysaccharide.

In the present study, the LPS levels in the serum from patients with hepatitis E virus (HEV)-related acute liver failure were examined and the apoptotic effects of the serum on human liver cells were investigated. In addition, the protective effects of antibody on serum-induced apoptosis in human liver cells were investigated.

## Materials and methods

### Reagents

The Quantitative Chromogenic Endpoint Tachypleus Amebocyte Lysate Endotoxin Detection kit was purchased from Xiamen Houshiji Ltd. (Xiamen, China). Anti-LPS antibody was purchased from MyBiosource (San Diego, CA, USA), which could recognize core polysaccharide. The Annexin V-FITC apoptosis detection kit was obtained from Nanjing KeyGen Biotech. Co. Ltd (Nanjing, China). RPMI-1640 medium, phosphate-buffered saline (PBS) and fetal bovine serum (FBS) were purchased from Gibco-BRL (Carlsbad, CA, USA).

### Serum collection and endotoxin determination

This study was approved by the ethics committee of Tongji Hospital (Wuhan, China). Whole blood samples from 13 patients with HEV-related acute liver failure and 13 normal individuals were collected. All participants signed a consent form approved by the ethics committee. Serum was separated from the blood by centrifugation at room temperature (3,000 × g for 10 min) and stored at −80°C. Serum endotoxin levels were measured using the endotoxin detection kit following the manufacturer’s instructions. Briefly, under aseptic conditions, 100 μl of the serum samples were incubated with 100 μl of the limulus amebocyte lysate at 37°C for 10 min, followed by incubation with the provided chromogenic substance at 37°C for 6 min. The absorbance was measured using a DU 730 spectrophotometer (Beckman Coulter Inc., Brea, CA, USA) at 545 nm following the addition of an azo reagent. The concentrations of endotoxins were calculated on the basis of a standard curve. Two serum samples with different concentrations of LPS were then used to prepare the serums with the mean concentration of LPS, which would be used in our subsequent experiment.

### Cell culture with acute liver failure serum

L02 human hepatic cell lines were preserved in a laboratory at the Department of Infectious Disease (Wuhan, China). The cells were maintained in RPMI-1640 medium supplemented with 10% FBS and incubated at 37°C in a 5% CO_2_ atmosphere. For experiments on cell apoptosis in different media, L02 cells were prepared and plated at 2×10^5^cells/well in 24-well plates in 500 μl medium and allowed to settle for 24 h. The cultures were later washed twice with warm PBS, and exposed to 20% (vol/vol) FBS, 20% (vol/vol) healthy serum or 20% (vol/vol) acute liver failure serum in RPMI-1640 medium (500 μl total volume). Following incubation for 20 h, cells were collected and prepared for the detection of apoptosis.

### Cell culture with antibody and acute liver failure serum

To measure the protective effects of the antibody on cells exposed to acute liver failure serum, L02 cells were plated at 2×10^5^cells/well in 24-well plates in 500 μl medium. The cells were seeded in six wells, allowed to settle for 24 h and the wells were divided into six groups: the FBS, healthy serum, acute liver failure serum, FBS + antibody, healthy serum + antibody and acute liver failure serum + antibody groups.

The cells were washed twice with warm PBS, and then exposed to medium containing 20% (vol/vol) FBS, 20% (vol/vol) healthy serum or 20% (vol/vol) acute liver failure serum. Simultaneously, 15 μl antibody (diluted 1:300) was added to the wells of the latter three groups.

Following incubation at 37°C in a 5% CO_2_ atmosphere for 20 h, the cells were collected and prepared for the detection of apoptosis.

### Measurement of apoptosis by flow cytometry

After treatment of the cells as described above, the cells were harvested, washed with PBS and stained using an Annexin V-FITC apoptosis detection kit. The total sample solution contained 500 μl binding buffer, 5 μl Annexin V-FITC and 5 μl propidium iodide. Following incubation at room temperature for 10 min, the cells were examined using a FACS Calibur flow cytometer (Becton Dickinson, Basel, Switzerland). Data were collected from 1×10^4^ cells in the gated region of each sample.

### Statistical analysis

Experiments were duplicated at least three times and the results were expressed as the mean ± standard deviation. One-way analysis of variance was used to analyze the statistical differences. Statistical analysis was carried out using SPSS software, version 13. 0 (SPSS Inc., Chicago, IL, USA). P<0.05 was considered to indicate a statistically significant difference.

## Results

### Clinical data of patients with HEV-related acute liver failure

Serum was collected from the blood of 13 patients with HEV-related acute liver failure and 13 normal individuals. The basic characteristics of the patients are listed in Table I.

### Serum LPS levels of patients with acute liver failure

**Figure 1:**
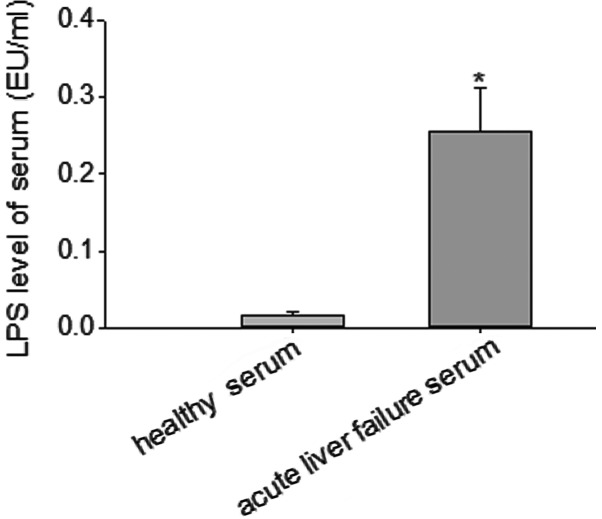
LPS levels in serum from patients with acute liver failure and healthy individuals. A quantitative tachypleus amebocyte lysate endotoxin detection kit with a chromogenic endpoint was used to measure the LPS levels. The results show a significant increase of LPS levels in the serum from patients with HEV-related acute liver failure compared with that of the healthy individuals. ^*^P<0.05 compared with healthy serum.

Serum was obtained from patients with HEV-related acute liver failure and normal individuals. A quantitative tachypleus amebocyte lysate-based endotoxin detection kit was used to measure the serum levels of LPS. The results indicated that the serum level of LPS in patients with acute liver failure was 0.26±0.02 EU/ml, which was significantly higher than that of the healthy individuals (P<0.05; Fig. 1).

### Proapoptotic effects of acute liver failure serum on cells

**Figure 2:**
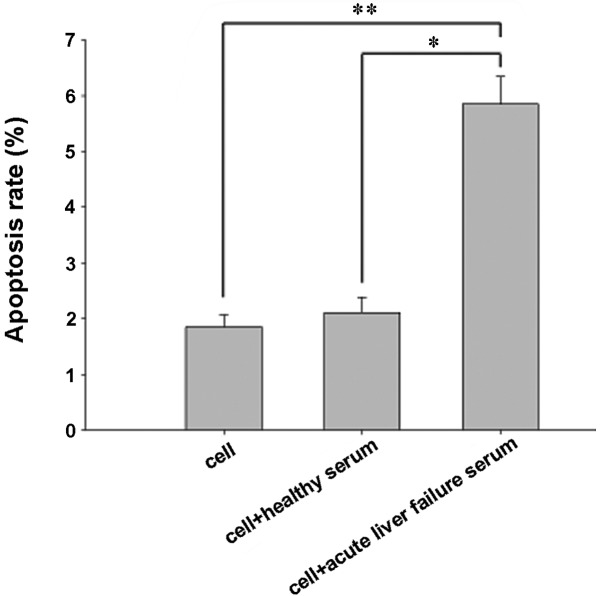
Apoptotic rate of cells was assayed by flow cytometry using an Annexin V-FITC apoptosis detection kit. L02 human hepatic cells were treated with RPMI-1640 medium supplemented with 20% FBS, healthy serum or acute liver failure serum for up to 20 h and then subjected to Annexin V-FITC and PI staining and flow cytometry. The acute liver failure serum triggered a significantly higher apoptosis rate than that in the FBS- and healthy serum-treated control groups. ^*^P<0.05 and ^**^P<0.05. FBS, fetal bovine serum; PI, propidium iodide.

To evaluate the proapoptotic effects of acute liver failure serum on human liver cells, flow cytometry was used to determine the apoptosis rate. The number of apoptotic cells at the lower and upper right of the flow cytometric analysis chart indicated the percentage of early and late apoptotic cells, respectively. The results indicated that the apoptotic rate of the cells incubated with acute liver failure serum was 5.83±0.42%, and that the apoptosis rate was significantly increased by the acute liver failure serum (P<0.05; Fig. 2).

### Protective effects of antibody against acute liver failure serum-induced apoptosis

**Figure 3:**
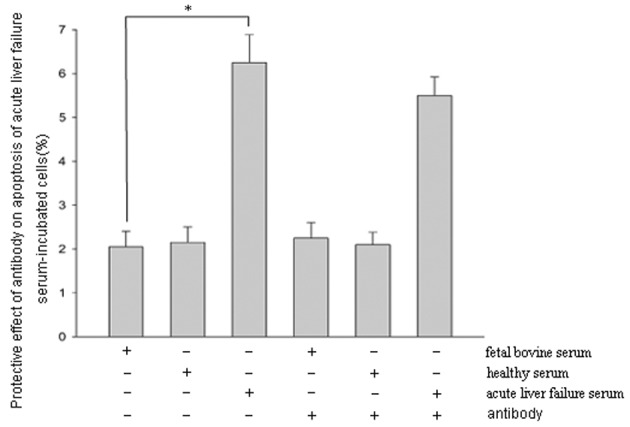
Flow cytometry analysis of liver cells treated with serum and antibody. L02 cells were incubated with acute liver failure serum and antibody for 20 h and flow cytometry was used to detect apoptosis. The results indicated that incubation of L02 cells with acute liver failure serum for 20 h caused a significant increase in the number of apoptotic cells compared with that of the control group (^*^P<0.05). However, the antibody did not significantly reduce acute liver failure serum-induced apoptosis of L02 cells.

To evaluate the protective effects of the antibody, cells were concurrently treated with the serum from patients with acute liver failure and antibody and examined by flow cytometry. The results showed that the rate of apoptosis in cells treated with acute liver failure serum alone was 6.21±0.67%. The rate of apoptosis decreased after 20 h of incubation with acute liver failure serum and antibody; however, the reduction was not statistically significant (Fig. 3).

## Discussion

The results of the present study indicate that the LPS levels in the serum of patients with HEV-related acute liver failure were significantly increased compared with those of healthy individuals, and that the acute liver failure serum was able to induce the apoptosis of liver cells. Anti-LPS antibody recognizing core polysaccharide demonstrated no significant protective effects on the apoptosis of cells treated with acute liver failure serum. The composition of the serum from patients with HEV-related acute liver failure is complicated and, the present study investigated only the serum levels of LPS and intervention against LPS using antibody.

Endotoxin syndrome occurs during the course of acute liver failure (2). In the present study, the acute liver failure in the patients was also associated with endotoxemia. It has been identified that the serum from patients with fulminant hepatic failure due to paracetamol overdose has the ability to induce apoptosis in primary hepatocytes (23). However, the mechanisms resulting in the apoptosis of liver cells are not fully understood. In a previous study, the plasma from patients with acute chronic liver failure did not significantly reduce cytochrome P450 mRNA expression levels in immortalized human hepatocytes (HepLi-2 cells); however, the ammonia removal and drug metabolism properties of the cells remained stable following incubation with plasma (24). Therefore, it may be speculated that the serum from patients with acute chronic liver failure does not result in apoptosis of HepLi-2 cells. In the present study, serum was collected from patients with HEV-related acute liver failure and the serum levels of LPS were evaluated. LPS has the ability to induce the apoptosis of liver cells without macrophages *in vitro*(25). Hence, it may be speculated that LPS in the serum may be the direct cause of the liver cell apoptosis observed in the present study; however, other substances that cause apoptosis of liver cells must not be excluded. The discrepancies in the findings concerning the effects of serum on liver cell survival and apoptosis may be attributed to several factors, such as differences in the cell lines, cell density and the sera from patients with liver failure due to a variety of causes.

The composition of acute liver failure serum is complex, containing various cytokines, bile salts and a number of other components. Each ingredient of the serum has different effects on liver cells, and the mechanisms of action have not been fully elucidated (26,27). Following the loss of liver synthesis and excretory functions, the levels of bile salts significantly increase in patients with liver failure. In initial studies, bile salts were viewed as intervention targets (28,29), however, the results were not satisfactory. LPS is the main component of the gram-negative bacterial cell wall and is associated with various biological effects (30,31). LPS consists of an outer membrane macromolecular complex of polysaccharides, lipids and proteins (32). Lipid A is the most conservative component of the LPS structure. The antibody against lipid A is considered to possess the most direct protective effects. In a study in which freeze-dried human plasma rich in anti-LPS IgG was used to treat septic shock, anti-LPS appeared to significantly reduce mortality and morbidity in patients with septicemia (33). However, the components of freeze-dried human plasma are complex. Human monoclonal IgM antibody that binds specifically to the lipid A domain of endotoxins has been shown to be safe and effective for the treatment of patients with sepsis and gram-negative bacteremia (34). However, an additional study found that anti-lipid A mAbs are not able to attenuate the toxic effects of LPS (22). The studies of the protective effects of lipid A antibody have provided greatly different results. Lipid A may have various epitopes and different antibodies against the epitopes may have correspondingly varied biological effects.

Core polysaccharide is a core component of LPS and following to the in-depth analysis of the lipid A antibody, scientists gradually shifted their attention to the core polysaccharide. In the present study, the core polysaccharide serum antibodies did not demonstrate significant protective effects against the actions of serum collected from patients with acute liver failure due to HEV infection. It was possible that the serum contained other harmful substances, which resisted the protective effects of the antibody.

In the present study, serum collected from patients with HEV-related acute liver failure induced apoptosis of human liver cells. This experiment was an in-depth study concerning the toxicity of the serum obtained from patients with acute liver failure. The effects of serum from patients with HEV-related acute liver failure on the survival and apoptosis rate of human liver cells were directly explored. However, the mechanisms by which acute liver failure serum affects liver cell survival and apoptosis have not been fully elucidated.

Bacterial LPS is an unavoidable aspect of liver failure development, which directly mediates the apoptosis of liver cells and aggravates the disease condition (25,35). However, in the present study, the antibody did not show protective effects and further study is required. The current study did not consider the complexity of the serum components and failed to take appropriate intervention measures. The aim of future studies is to analyze the different components of acute liver failure serum, as well as the mechanisms of cell survival and apoptosis affected by these components. This is likely to assist in clarifying the pathogenesis of acute liver failure and the therapeutic effects of LPS antibody on acute liver failure.

## Figures and Tables

**Table I:** Baseline characteristics of patients with HEV-related acute liver failure and healthy individuals.

Characteristics	Healthy (n=13)	Acute liver failure (n=13)
Gender		
Male	8	10
Female	5	3
Age (years)	34.5±7.1	39.5±7.3
ALT (U/l)	30.6±5.5	694.9±331.7
AST (U/l)	29.0±4.6	455.8±217.7
TBIL (μmol/l)	11.2±3.2	258.8±66.1
PTA (%)	93.7±5.4	29.4±4.3

Measurement data are the mean ± standard deviation. HEV, hepatitis E virus; ALT, alanine aminotransferase; AST, aspartate aminotransferase; TBIL, total bilirubin; PTA, prothrombin time activity.
